# Social isolation and metabolic syndrome among community dwelling, low-income older adults: What we need to know!

**DOI:** 10.1093/geroni/igaf122.2789

**Published:** 2025-12-31

**Authors:** Karen Bullock, Barbara Mendez Campos, Terrance Ruth, Kim Stansbury

**Affiliations:** Boston College, Chestnut Hill, Massachusetts, United States; University of Tennessee Knoxville, Knoxville, Tennessee, United States; North Carolina State University, Raleigh, North Carolina, United States; North Carolina State University, Raleigh, North Carolina, United States

## Abstract

During the COVID-pandemic, social determinants of health were brought to light for many gerontologists in ways that had not been given close attention in older adult communities before. Such non-medical factors that influence a person’s health and wellbeing can include socio-economic status, education, social support, healthcare access, transportation and more. This study explores social isolation as a factor contributing to health and well-being among older adults. Studies show physical and emotional association between human connection and the clustering of biological factors, also known as metabolic syndrome. Such clustering of health conditions such as cardiovascular disease, arthritis, kidney disease, several types of cancers, schizophrenia and other mental health disorders constitute metabolic syndrome, which is more prevalent among older adults of racial and ethnic groups that are known to have the highest rates of morbidity. Furthermore, low educational attainment and advanced age have been associated with higher reports of social isolations and loneliness. However, less is known about the incidences of chronic illness among an older adult population living in low-income senior housing communities and social isolation, which can be associated with loneliness, which is also risk factor associated with well-being. Preliminary data is presented to highlight the importance of understanding and intervening with community-dwelling older adults to help keep them thriving and aging.

